# Response of the TEROS 12 Soil Moisture Sensor under Different Soils and Variable Electrical Conductivity

**DOI:** 10.3390/s24072206

**Published:** 2024-03-29

**Authors:** Athanasios Fragkos, Dimitrios Loukatos, Georgios Kargas, Konstantinos G. Arvanitis

**Affiliations:** Department of Natural Resources Management and Agricultural Engineering, Agricultural University of Athens, 75 Iera Odos Str., 11855 Athens, Greece; athfrag@aua.gr (A.F.); dlouka@aua.gr (D.L.); karvan@aua.gr (K.G.A.)

**Keywords:** electrical conductivity, apparent dielectric permittivity, soil moisture, TEROS 12, calibration, microcontroller-based measurements, crop production, resource preservation

## Abstract

In this work, the performance of the TEROS 12 electromagnetic sensor, which measures volumetric soil water content (θ), bulk soil electrical conductivity (σ_b_), and temperature, is examined for a number of different soils, different θ and different levels of the electrical conductivity of the soil solution (EC_W_) under laboratory conditions. For the above reason, a prototype device was developed including a low-cost microcontroller and suitable adaptation circuits for the aforementioned sensor. Six characteristic porous media were examined in a θ range from air drying to saturation, while four different solutions of increasing Electrical Conductivity (EC_w_) from 0.28 dS/m to approximately 10 dS/m were used in four of these porous media. It was found that TEROS 12 apparent dielectric permittivity (ε_a_) readings were lower than that of Topp’s permittivity–water content relationship, especially at higher soil water content values in the coarse porous bodies. The differences are observed in sand (S), sandy loam (SL) and loam (L), at this order. The results suggested that the relationship between experimentally measured soil water content (θ_m_) and ε_a_^0.5^ was strongly linear (0.869 < R^2^ < 0.989), but the linearity of the relation θ_m_-ε_a_^0.5^ decreases with the increase in bulk EC (σ_b_) of the soil. The most accurate results were provided by the multipoint calibration method (CAL), as evaluated with the root mean square error (RMSE). Also, it was found that ε_a_ degrades substantially at values of σ_b_ less than 2.5 dS/m while ε_a_ returns to near 80 at higher values. Regarding the relation ε_a_-σ_b_, it seems that it is strongly linear and that its slope depends on the pore water electrical conductivity (σ_p_) and the soil type.

## 1. Introduction

The accurate estimation of volumetric soil water content (θ) helps in the efficient management of irrigation water to obtain high crop production but also in its low consumption In this regard, technologies like electronics, sensors, automation, networking and artificial intelligence have the potential to contribute to the transformation of the agri-food sector and provide systems capable of efficient processing, decision making, action and accurate real-time sensing [1,2,3,4]. The adaptation of irrigated agriculture to the new conditions is imperative in order to ensure that there will be sufficient quantities of water of suitable quality to support the functions of the natural environment both today and in the future.

Modern dielectric capacitance sensors, which are characterized by a low operating frequency (*f*), usually less than 100 MHz, provide the possibility of continuous recording of θ. Capacitance sensors determine apparent soil dielectric permittivity by measuring the charge time of a capacitor (i.e., the soil-probe system) for a given voltage. Due to a sensor’s low operating frequency, determination of the apparent dielectric permittivity is affected by bulk soil electrical conductivity (σ_b_); as a result, the estimation of θ is not always accurate [5,6]. Thus, the accurate measurement of θ requires the special calibration of the device, i.e., the correlation of the (θ) and the apparent dielectric permittivity (ε_a_) with the sensor measurements. Several of these sensors also measure other soil properties such as soil temperature (T) and bulk soil electrical conductivity (σ_b_) [7,8].

Whereas many commercial sensors provide calibration equation, which combine the square root of the apparent dielectric permittivity with the soil water content, several researchers report the possibility of further improving this specific manufacturer equation [9,10]. Toward this direction, the main objective of this study was to evaluate the accuracy of the empirical calibration equations for the TEROS 12 sensor, as given by the manufacturer. An important question is whether or not the primary (i.e., uncalibrated) measurements, as provided by the sensor for different soil types and for different salinity conditions, give the expected values, when put into the manufacturer calibration equations, for (θ) and (ε_a_). The detailed and independent evaluation of this sensor aims to provide fertile ground for improving its overall performance toward a better exploitation of its potential. A prototype device, utilizing widely available electronics was developed for facilitating the raw measurement process.

## 2. Background Work and Motivations

Almost all soil sensors available today use ε_α_ as a basis for determining θ. This approach is based on the highly apparent dielectric permittivity of water (ε_α_ = 80) as opposed to soil solids (ε_α_ = 2–5) or air (ε_α_ = 1). Topp et al. [11] demonstrated that dielectric measurements could be used to accurately measure the (θ) using the TDR (Time Domain Reflectometry) method. The large difference between the values of the apparent dielectric permittivity of water and the other constituents of soil makes the apparent dielectric permittivity sensitive to θ.

The TDR method is able to accurately calculate a material’s dielectric permittivity from wave propagation, because of the fact that a material’s permittivity and its water content are strongly related, as it was also presented by Hoekstra and Delaney [12] and Topp et al. [11]. By measuring electromagnetic pulse reflection time, we calculate θ indirectly by determining the apparent dielectric permittivity. The time required for a high-frequency electromagnetic wave (300–1000 MHz) to traverse the length of a waveguide is measured in order to assess the ε_α_, which is directly related to the response time of the wave. Although TDR is a reliable method for verifying soil or substrate θ, its expensive cost has led in the development of alternative dielectric sensors technologies (capacitance, impedance, and transmission line oscillators), which are cheaper and do not rely on complicated waveform analysis. The capacitance sensors determine the charging time of the sensor capacitor depending on apparent dielectric permittivity. The sensor TEROS 12 uses the enclosing soil surrounding the three needles as the dielectric of the capacitor, from which it measures its charge time and output a raw voltage based on the substrate ε_a_.

The dielectric methods have been extensively studied and constitute an accepted measurement technique as it does not disorder the ground and is suitable for automation and remote sensing. Furthermore, the improvement of θ estimation has been attempted, taking into account the effect of (σ_b_) on apparent dielectric permittivity [7,13,14,15,16].

The response of a dielectric material to electromagnetic fields is expressed by a complex function [17].

ε* = ε′ − jε″,(1)

of which the real part (ε′) expresses the storage of electric energy in the medium while the imaginary part (ε″) expresses its diffusion (loss factor) in the means of propagation, with $j=\sqrt{-1}$. If we divide the relation (1) by the dielectric constant of the vacuum ε_ο_ = 8.85∙10^−12^ Fm^−1^, we obtain its conversion to the relation (2)

ε*_r_ = ε′_r_ − jε″_r_, (2)

where ε*_r_ is relative apparent permittivity, the real part ε′_r_ is the dielectric constant that expresses how much energy can be stored by the electromagnetic field imposed and is mainly related to soil moisture θ as well as to the spatial distribution of the soil’s liquid phase while the imaginary part ε″_r_ expresses the dielectric losses that occur when the mechanism of polarization of water in the soil does not follow exactly the changes of the applied field. Polarization is the rearrangement of charges inside the material, to compensate the electric field (i.e., the positive and negative charges as they move in different directions). These relations are critical because dielectric permittivity is directly related to both ε′_r_ and ε″_r_ for almost all soil water sensors.

The dielectric constant and dielectric losses vary with applied frequency *f* and their detailed study should focus on the frequency range operated by a soil sensor. The real part of the relative apparent permittivity appears to take the largest values at low operation frequencies and generally decreases with increasing frequency.

Other soil factors that affect apparent permittivity are in general: pore and specific surface area distributions, grain and pore shape and size, organic matter content, fabric and structure of soil, porosity, chemical composition of the soil solution, temperature, and bulk soil electrical conductivity (σ_b_) [18,19,20,21]. Due to the number of these parameters we focused to evaluate the effects of (σ_b_) on the sensor performance and estimate if a specific calibration procedure is needed.

For the case of TDR, it has been shown that dielectric permittivity can be related to θ with reasonable accuracy, and thus θ can be calculated from it, for a wide variety of soils, using a single calibration equation [11]. Alternatives to empirically derived calibrations are often based on the dielectric mixing model. Ferre and Topp [22] indicated that soil water content can be calculated from ε_a_ using a simple regression formula that relates θ to ε_a_, using the form of Equation (3), where a and b are suitable fitting parameters.

(3) $$\theta =a\sqrt{\varepsilon_{a}}+b$$

In this regard, for the TDR measurements, Topp and Reynolds [23], using the general form of Equation (3) with parameter α equal to 0.115 and parameter b equal to −0.176, have shown that the derived formula, Equation (4),

(4) $$\theta =0.115\sqrt{\varepsilon_{a}}-0.176$$

is effectively equivalent to the third-order calibration equation suggested by Topp et al. in 1980 [11] and it deviates less than 0.01 m^3^ m^−3^ from it, for θ values calculated over a range from 0.05 to 0.45 m^3^ m^−3^.

The existence of a corresponding linear relationship θ-ε^0.5^ has been demonstrated for many dielectric sensors. But there is a big difference in the values of parameters a, b (Equation (3)) compared to those values for the case of TDR (Equation (4)) [7,24,25,26,27,28]. These values are affected by the type of soil, the operating frequency of the sensor, the temperature, the bulk soil electrical conductivity and the special characteristics of the sensor. Nasta et. al., [8] examined two soils (loamy and clay) and showed that the application of soil-specific temperature correction is highly recommended, if the TEROS 12 is installed in regions with pronounced seasonal variations in soil temperature.

However, the question remains open whether a linear relationship θ-ε^0.5^ is valid in many types of soil and whether the coefficients of the linear relationship differ depending on the type of soil. We also investigate whether the accuracy of the sensors is affected and how by changes in soil salinity.

## 3. Materials and Methods

### 3.1. Soil Sensor Characteristics

The TEROS 12 sensor from METER Group AG indirectly measures the volumetric water content in the soil as well as the apparent dielectric permittivity of the porous medium by emitting an electromagnetic wave frequency of 70 MHz, and also measures temperature using a thermistor which is in the center needle and bulk electrical conductivity. It has three stainless steel needles (i.e., instead of the two that the TEROS 10 sensor has), each 5.5 cm long, and is compatible with most data recording systems. The sensor delivers an electromagnetic wave to the needles that are charged depending on the dielectricity of the material. The charging time is proportional to the dielectric current and θ of the substrate. The resulting measurement volume is approximately 1010 cm^3^. The TEROS 12 sensor comes pre-calibrated for mineral soil and has the following specifications volumetric water ranges from 0 to 0.70 m^3^ m^−3^, accuracy is ±3% in mineral soils with standard factory calibration when the electrical conductivity of the solution is EC < 8 dS/m, the temperature measurement range is −40 to 60 °C with a resolution of 0.1 °C and the measurement accuracy is ±1 °C. According to its manual [29], the electrical conductivity measurement range of TEROS 12 is 0–10 dS/m, with a resolution of 0.001 dS/m and a measuring accuracy of 5%. The TEROS 12 sensor is known for its low energy consumption (3.5 mA in the operative mode and 0.03 mA in the sleep mode).

According to the TEROS 12 manual for third-party data loggers, each fresh raw θ sensor value (RAW) should be put into the proper calibration equation and the corresponding value be extracted. The equations for calibrating and deriving the real values from the raw values are given by the following equations, for soil moisture:

θ (m^3^ m^−3^) = 3.879 ∙ 10^−4^ ∙ RAW − 0.6956, (5)

and for the dielectric permittivity:

ε_a_ = (2.887 ∙ 10^−9^ ∙ RAW^3^ − 2.080 ∙ 10^−5^ ∙ RAW^2^ + 5.276 ∙ 10^−2^ ∙ RAW − 43.39)^2^, (6)

As a remark, for the bulk conductivity provided by the sensor, the original raw values were following two different formats (i.e., in dS/m and in μS/cm) according to their magnitude, and thus a division of the raw values by 1000 was necessary for the small quantities (expressed in μS/cm), a practice that its necessity was also observed by Tadaomi Saito et al. [30].

### 3.2. Technical Arrangements for Data Collection and Processing

In order to exploit the data provided by the TEROS 12 soil sensor, a cost-effective microcontroller was properly wired and programmed, based on the ATmega32U4 chip. This microcontroller is a 3.3 V logic unit and is equipped with analog input pins and digital input/output pins. It also has a USB port for easy connectivity and programming purposes via a conventional computer/laptop. For the latter task, the Arduino IDE environment has been the preferred option, which also provides fast and easy debugging and monitoring options via its Serial Monitor and the Serial Plotter components.

**Figure 1:**
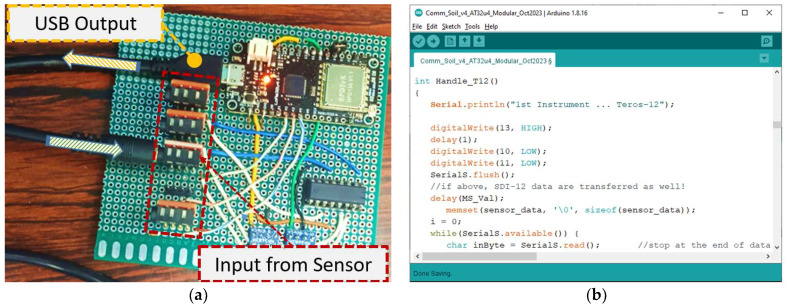
Technical arrangement details supporting the data acquisition process: (**a**) hardware; (**b**) software.

Prior to interfacing the soil instrument with the prototype measuring equipment, a thorough study of the sensor physiology was necessary. More specifically, according to the manufacturer’s instructions for TEROS 12, a voltage of 4 to 15 V DC should be supplied while its input can tolerate signals from 2.8 to 5.0 V. The digital output of this sensor is compatible with the 3.3 V logic. The physical connection with the microcontroller is addressed via a custom measuring circuit able to host the 3.5 mm stereo plug connector the soil sensor has. Special care should be taken as the TEROS 12 in its default settings, after warming up, transmits a fresh triplet of soil volumetric water content (VWC), temperature and bulk conductivity values according to the simple serial protocol called DDI; and, after this, it enters the more complex serial communication mode called SDI-12. For taking the measurements that this research is using, the DDI method is adequate, and thus the necessary code was written in the microcontroller memory, according to the timing diagram provided by the sensor manufacturer. A voltage converter raised the voltage from 3.3 to 5 Volts to supply the sensor, and a multiplexer directed the data to the desired serial input line, in case of more than one instrument connected with the same acquisition unit. The microcontroller was also responsible for periodically powering on and off the sensor, every 10 s. Technical arrangement details supporting the data acquisition process are shown in Figure 1.

The RAW data acquired from the TEROS 12 via the microcontroller were applied in the calibration equations (implemented on the microcontroller) to calculate the soil moisture and the dielectrical permittivity according to Equations (3) and (4), respectively. The latter values, along with the original triplet (i.e., the VWC, temperature and the bulk conductivity values) were forwarded to a laptop, connected to the microcontroller via a USB connection, for storage and further processing and/or visualization, typically utilizing the MS Excel application. Sensor-to-sensor variability effects were ignored in our work by crudely assuming limited sensor-to-sensor variability, similar to Nasta et al. in [8].

### 3.3. Measurement in Soils

The experiments were conducted using six different inorganic soil types. In greater detail, 5 of the 6 soil samples being mentioned were collected from agricultural areas and more specifically from the Prefecture of Argolida [31]. The 6th one, i.e., the sand sample, is used as part of artificial substrates in floriculture. All experiments were performed at constant temperature (23 ± 1 °C) to avoid temperature effects. All the air-dried soils were ground sieved to reach particle size ≤ 2 mm and were later put in the oven to calculate the initial air-dried soil moisture, via measuring their weight loss. Calculation of the dry bulk density of every sample was performed to convert the moisture from a mass base to a volume base. The corresponding soil properties are presented in Table 1. These soils were chosen because they exhibit a wide variety of properties. As can be seen from Table 1, the clay content ranges from 0% to 48%. However, the study of the characteristics of the pore structure and the type of clay in each soil is under investigation [32]. Soils samples of sand, clay, loam, sandy loam, sandy clay loam, and silty clay loam were used, in which an increase in θ took place in a strictly defined manner up to the saturation point. In particular, an air-dried soil sample volume of 1500 mL was used, to which 75 mL of tap water (EC = 0.28 dS/m) was added each time in order to increase the actual water content (θ_m_) each time by θ = 0.05 m^3^ m^−3^. Pressed with a 0.20 kg hammer, the soil material of different water content levels was aimed to achieve uniform density distribution in the soil sample. The θ_m_ and dry bulk density of the soils were determined again by weighing and oven drying at the end of the experiment. In addition, measurements were taken in an oven-dried soil sample (θ = 0 m^3^ m^−3^). Via this step sequence, a sufficient number of ε_a_, σ_b_ and θ values for all the soil types examined were provided. For each value of θ_m_, four measurements of the RAW and T, σ_b_ data acquired from the TEROS 12 via the microcontroller were applied in the calibration equations (implemented on the microcontroller) and then the average was calculated. This was considered to be appropriate, with the scope of detecting any errors during the measurements.

**Figure 2:**
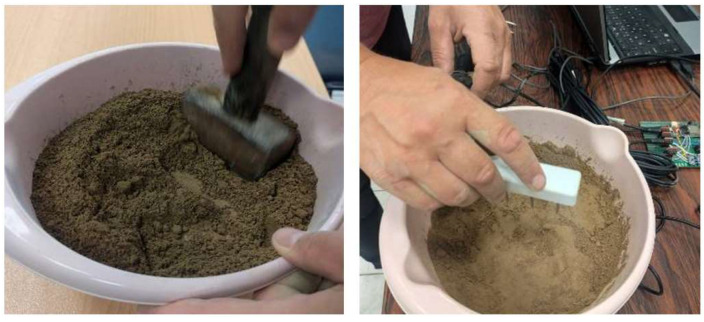
Characteristic instances from the soil preparation and measurement process.

At the same time, it was ensured that the walls of the container are more than 3 cm apart, which the manufacturer requires at least in order to have no effect on the measurements and not affect the readings of the sensor. The sensor was vertically inserted into the soil samples and measurements were taken. Figure 2 depicts characteristic instances from the soil preparation and measurement process via the TEROS 12 sensor.

### 3.4. Salinity Effects in Water Solutions and Soils

To investigate the effect of salinity on the performance of TEROS 12, experiments with increased EC_w_ were carried out in aqueous solutions and in soils. Initially, measurements in water solutions with increasing electrical conductivity values (EC_w_) from 0 to 20 dS/m by adding potassium salt (i.e., potassium chloride—KCl) to deionized water were made in order to investigate TEROS 12 sensitivity and response to EC_w_ changes. The sensor was inserted vertically into water solutions and the output readings were determined in different EC_w_. This experiment is a guide for the effect of EC_w_ on the stability of apparent dielectric permittivity measurements.

Moreover, the experimental procedure mentioned in the part «3.3 Measurements in soils» was used in order to evaluate the sensor’s sensitivity to salinity for four soil materials (sand, sandy loam, loam, clay). In addition to the KCl solutions of EC_w_ = 0.28 dS/m, similar experiments were carried out with KCl solutions of EC_w_ 3, 6 and 10 dS/m. For sandy clay loam and silty clay loam the response of the TEROS 12 was tested and evaluated only for EC_w_ = 0.28 dS/m.

### 3.5. Prediction of Pore-Water Electrical Conductivity

As a final step in this study, the prediction of soil pore water electrical conductivity (σ_p_) by utilizing TEROS 12 sensor measurements has been attempted. For this purpose the σ_p_ prediction model, of the Hilhorst [33] model was used. This model is connecting ε_a_, σ_b_ and σ_p_ through the equation

(7) $$\;\sigma_{p}=\frac{\varepsilon_{p}\;\sigma_{b}}{\varepsilon_{a}-\varepsilon_{0}}$$

where ε_p_ is the apparent dielectric permittivity of the pore water, considered equal to that of pure water (80.3 at 20 °C), and ε_0_ is the y-intercept of the line ε_a_ = f(σ_b_). Using Equation (7), σ_p_ can be calculated from a simultaneous measurement of ε_a_ and σ_b_.

Hilhorst [33] found that ε_0_ was in the range of 1.9–7.6. The value of 4.1 was proposed to be used as a mean value for all soil types.

### 3.6. Performance Evaluation Criteria

To evaluate the efficiency of the various calibration equations, the root mean square error (RMSE) and the correlation coefficient R^2^ were used as evaluation criteria.

(8) $$RMSE=\sqrt{\frac{1}{n}\sum_{i=1}^{n}{{(O}_{i-}P_{i})}^{2}}$$

(9) $$R^{2}=\frac{\sum_{i=1}^{n}{{(O}_{i}-P_{i)}}^{2}}{\sum_{i=1}^{n}{(O}_{i}-{\bar{O})}^{2}}$$

where O is the observed value, $\bar{O}$ is the mean observed value, P is the predicted value of soil water content, *i* is the counter for data pairs and n is the total number of different pairs of observed–predicted values. Good prediction requires data with low bias and error, gibing a low root mean square error. When RMSE approaches 0, this means we have very good prediction, while when it approaches 1, we have a prediction about the same as the average value. The correlation coefficient R^2^ varies between 0 and 1; when it approaches 0, there is no correlation, while we have a very strong correlation when it approaches 1. In intermediate values between 0 and 1, we have a weak, mean or strong correlation.

## 4. Results and Discussion

### 4.1. Salinity Effects in Liquid Solutions

**Figure 3:**
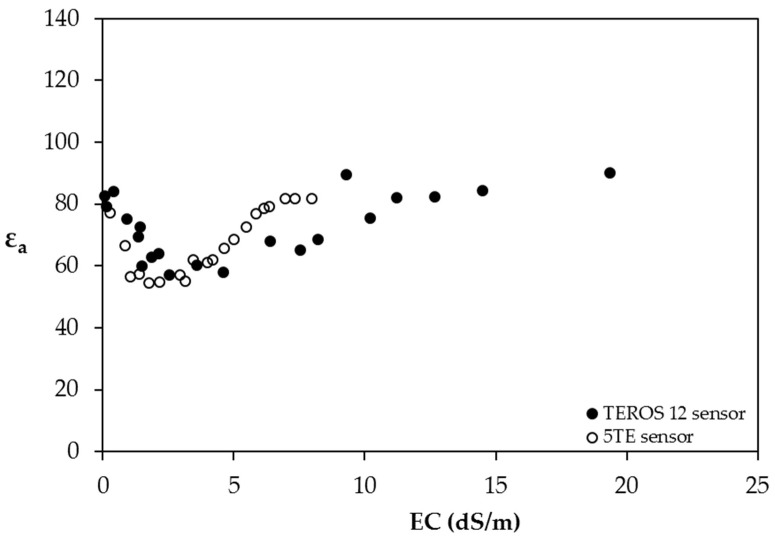
The ε_a_ values against EC_w_ in aqueous KCl solutions for the TEROS 12 and the 5TE sensors.

In Figure 3, the sensor (ε_a_) response to increasing EC_w_ in water is shown, where EC_w_ = σ_b_ is the complex. The ε_a_ values, which should be between 78 and 80, decrease at approximately the value of 60 with increasing salinity to the value of 2.5 dS/m, stabilizing at approximately the value of 60 between the values of 2.5 and 6 dS/m, then increasing up to 80 with increasing salinity to the value of 10 dS/m and then remaining constant at approximately 80. This behavior is contrary to what is reported by Seyfried and Murdock [25] for the Hydra Probe sensor, by Hilhorst [30] for the Sigma probe sensor, by Kargas et al. [6] for the WET sensor and Kargas and by Soulis [7] for the CS655 sensor, which operate at low frequencies and are not affected by the increase in salinity up to 3 dS/m. As observed, there is a high sensitivity of the TEROS 12 to relatively low σ_b_ values. This behavior is contrary to what was expected due to the effect of the low operating frequency of TEROS 12 and may only be attributed to the sensor design. Rosenbaum et al. [34], Schwartz et al. [15] and Kargas et al. [6] reported similar sensitivity abnormality studying the 5TE sensor behavior.

### 4.2. Soil-Specific Calibration

**Figure 4:**
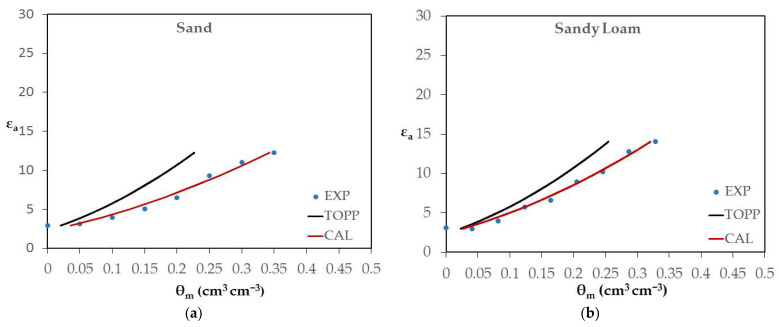

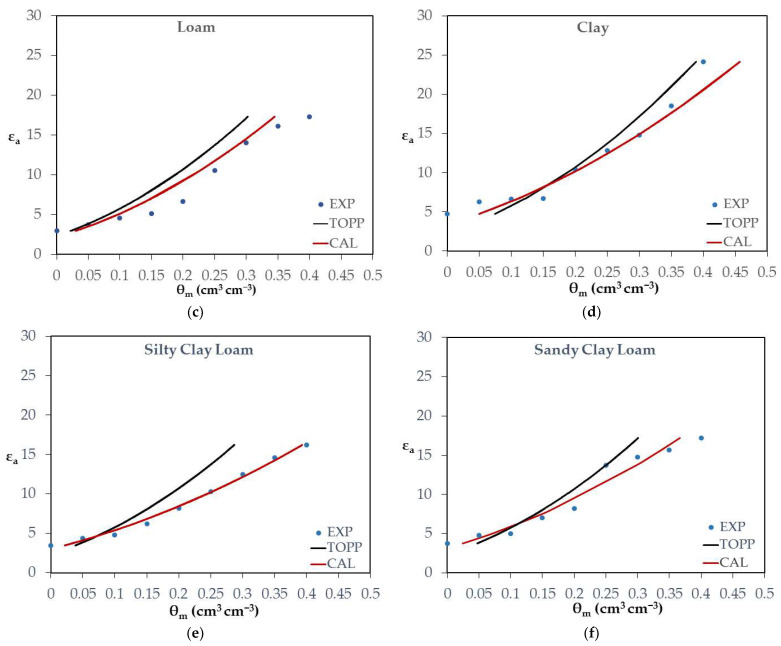
The apparent dielectric permittivity (unitless) and soil water content (in cm^3^ cm^−3^) relationship (i.e., ε_a_-θ_m_) for characteristic soil samples and for a salinity of EC_w_ = 0.28 dS/m (blue points). More specifically, the soil cases were: (**a**) sand; (**b**) sandy loam; (**c**) loam; (**d**) clay; (**e**) silty clay loam; (**f**) sandy clay loam. Two other curves are also depicted per soil type. The first curve (black line) expresses the relationship ε_a_-θ, where θ is calculated by Equation (4) (TOPP curve) and the second curve (red line) expresses the relationship ε_a_-θ, where the θ is calculated by soil-specific CAL calibration equation (CAL curve).

To better study the TEROS 12 behavior, further experiments were conducted using soil samples instead of liquid solutions. The corresponding results are depicted in Figure 4. In greater detail, the relationship between the apparent dielectric permittivity (unitless) and the actual soil water content, i.e., ε_a_-θ_m_, was determined (as depicted by blue points in Figure 4a–f—EXP) for characteristic soil samples and for a salinity level of EC_w_ = 0.28 dS/m. The soil samples were sand, sandy loam, loam, clay, silty clay loam and sandy clay loam. For better comparison purposes, two other curves are also depicted in each soil case figure. The first curve (black line) expresses the relationship ε_a_-θ where θ is calculated by Equation (4) (TOPP curve) and the second curve (red line) expresses the relationship ε_a_-θ where the θ is calculated by the soil-specific CAL calibration equation (CAL curve).

By inspecting the results in Figure 4, it is inferred that TEROS 12 always gives lower values for ε_a_, compared to the ones derived via the Topp equation for the same θ_m_ level. The greatest difference is observed in sand, i.e., 12 units. In sandy loam, the difference becomes slightly lower, i.e., 10.5 units, while it further drops to 5 units in loam. In clay, it is observed that the values of ε_a_, given by TEROS 12, are closer to the Topp values.

These results are reflected in the calibration parameters shown in Table 2, where both a and b parameters are substantially greater than the Topp equation ones for all soil types being used. These results are quite unexpected because the TEROS 12 *f* is only 70 MHz, much smaller than that for TDR. As was shown from Table 2, the θ_m_-ε^0.5^ relationship is strongly linear in all soils. Additionally, it appears that each soil type has a separate calibration equation. Thus, a single calibration equation could not work for all soil types. The corresponding R^2^ values as well as the σ_b_ readings obtained by the TEROS 12 sensor at saturation are also presented in Table 2. More specifically, the R^2^ values range from 0.952 to 0.989.

The corresponding RMSE values for the θ predictions obtained by the specific calibration method and the manufacturer calibration are presented in Table 3. 

From the results, it is observed that the CAL procedure has much smaller RMSE values compared to the manufacturer calibration equation. More specifically, the RMSE values for the CAL calibration method range from 0.013 to 0.046 m^3^ m^−3^, while the RMSE values for the manufacturer calibration range from 0.027 to 0.098 m^3^ m^−3^. Using CAL parameters, the average RMSE was reduced by almost 50%. These results indicate that the CAL is an effective soil-specific procedure.

### 4.3. Salinity Effects in Soils

The experimental arrangements described in Section 3.4 were applied in order to investigate the sensor’s response and sensitivity to the indicative salinity levels of 0.28, 3, 6 and 10 dS/m and for four soils, namely sand, sandy loam, loam and clay. In this case, KCl solutions with known EC_w_ were added to obtain predetermined θ_m_ levels from dry to saturation.

**Figure 5:**
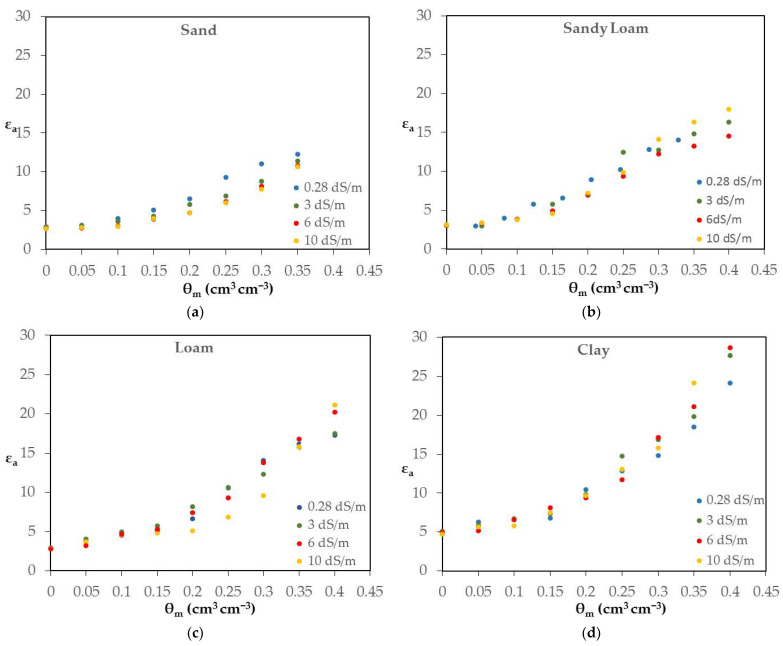
The apparent dielectric permittivity (unitless) and soil water content (in cm^3^ cm^−3^) relationship (i.e., ε_a_-θ_m_) for specific salinity levels and for the characteristic soil samples: (**a**) sand; (**b**) sandy loam; (**c**) loam; (**d**) clay.

As obvious from Figure 5a, when EC_w_ increases, the ε_a_ value for the same θ_m_ decreases instead of increasing, because of the low-frequency operation of TEROS 12.

The greatest values of the coefficients of the equation θ_m_-ε^0.5^ in sand are observed in the large values of bulk electrical conductivity σ_b_ because the values ε_a_ decrease with the increase of σ_b_ (max σ_b_ sand = 1.870 dS/m) (Table 2). This behavior is similar to the corresponding in the solutions with increasing salinity level. It appears that the unexpected behavior of the TEROS 12 sensor was related to its response to σ_b_, which was highly nonlinear and inverse, from negative to small σ_b_ to positive to large σ_b_. Tadaomi Saito et al. [30] reported a similar behavior of the TEROS 12 sensor.

On the contrary, in the clay soil (Figure 5d), which is most affected by ε″, the calibration relationships are closer to the Topp equation than the sand especially in the higher EC_w_ value. In the case of clay soil, the decrease in ε_a_ may be compensated from its increase because of the effect of ε″. Consequently, the coefficients of the calibration equations have a lower value compared to those of sand.

Sandy loam and loam soils (Figure 5b and Figure 5c, respectively) exhibit an intermediate behavior. More specifically, up to a θ_m_ value of approximately 0.3 m^3^ m^−3^, the values of ε_a_ corresponding to EC_w_ = 0.28 dS/m are higher than those corresponding to EC_w_ = 10 dS/m, while the tendency reverses above this value of θ_m_.

However, even though this complication appears, a strong linearity of the θ_m_-ε^0.5^ relationship is again observed with increasing σ_b_. As can be seen from Table 2, the value of R^2^ is always greater than 0.925. Only in the case of loam at EC_w_ = 10 dS/m, the R^2^ value is relatively small (R^2^ = 0.869).

Nevertheless, it is demonstrated that the linearity decreases with the increase in σ_b_ values. The most characteristic case is loam, where R^2^ = 0.961 in EC_w_ = 0.28 dS/m, whereas R^2^ = 0.869 in EC_w_ = 10 dS/m.

### 4.4. Prediction of Pore-Water Electrical Conductivity

**Figure 6:**
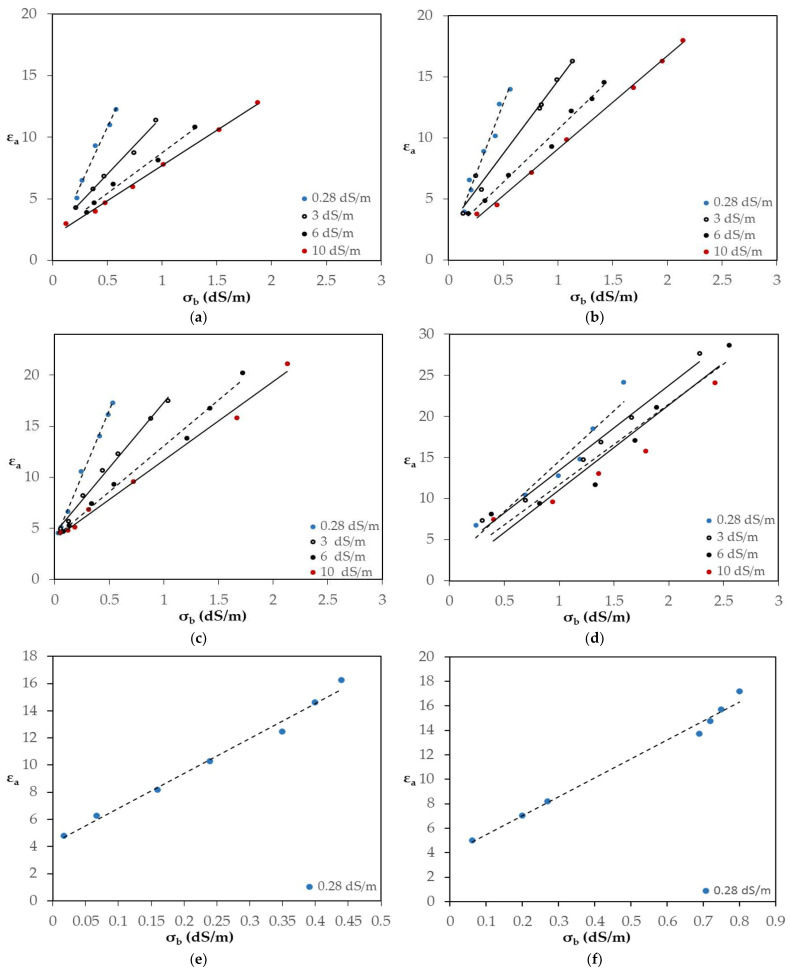
Τhe relationship ε_a_-σ_b_ for various EC_w_ levels and for the characteristic soil types: (**a**) sand; (**b**) sandy loam; (**c**) loam; (**d**) clay; (**e**) silty clay loam; (**f**) sandy clay loam. For the last two types of soil, the relation ε_a_-σ_b_ refers only to EC_w_ = 0.28 dSm^−1^.

Τhe ε_a_ measurements (for different EC_w_ levels and for each of the characteristic soil samples) were plotted against the corresponding σ_b_ measurements. More specifically, experiments involved EC_w_ levels equal to 0.28, 3, 6, 10 dS/m, for the sand, sandy loam, loam and clay soil types. Furthermore, two additional experiment sets were conducted for silty clay loam and for sandy clay loam, with a salinity level of EC_w_ = 0.28 dS/m. The resulting curves are shown in Figure 6a–f, respectively.

As can be clearly seen, in Figure 6, the slope of the line is salinity dependent and decreases when the EC_w_ increases. In sand at EC_w_ = 0.28 dS/m, the slope of the line is 19.360, whereas at EC = 10 dS/m the corresponding slope is approximately three times lower (5.729). Also, it seems that the value of the slope is affected by the soil type. Concerning the value of ε_0_, it appears that is much lower than the 4.1 value with a higher value, 3.1, for sand, sandy loam and clay soils. In the loam, silty clay loam and sandy clay loam soil types, the values of ε_0_ are approximately 4.

The obtained results using the TEROS 12 sensor revealed that the ε_a_-σ_b_ relationship is strongly linear in all salinity level cases, since the values of R^2^ ranged between 0.878 and 0.997, as shown in Table 4.

From Table 5, it appears that the Hilhorst model predicts values of σ_p_ considerably higher than those of the wetting solution EC_w_ values. However, if the values of the electrical conductivity of the extract’s saturation paste (EC_e_), which were measured for each soil (Table 5), are taken into account, the soils initially contained relatively high amounts of salts.

Considering that the initial values of σ_p_ are approximately twice the values of EC_e_ in the field capacity, the overestimation of σ_p_ is not that great. More specifically in the loam soil for EC_w_ = 3 dS/m, the value of σ_p_ = 6.21 dS/m. This value is approximately equal to the sum of EC_w_ plus twice the value of EC_e_. Consequently, the Hilhorst model combined with the TEROS 12 sensor satisfactorily approximates the salinity level in soils.

## 5. Conclusions

The performance of the TEROS 12 soil moisture sensor was evaluated under six different soil types and four salinity levels under laboratory experiments. The behavior of the TEROS 12 sensor is complex as the EC increases. In liquids with increasing EC, there is an anomalous behavior of the TEROS 12 sensor. This behavior is related to its response to σ_b_, which is highly nonlinear and in the reverse direction, from negative at small σ_b_ to positive at large σ_b_.

The θ_m_-$\sqrt{\varepsilon }$ relationship was strongly linear in all examined cases but linearity was reduced at the high salinity level. The CAL calibration procedure, as evaluated with the RMSE, was effective for all soils. Additionally, it appears that each soil type has a separate calibration equation. The above underlines the importance of carrying out a specific laboratory experiment for the correct conversion of apparent dielectric permittivity to soil water content (θ), especially under a regime of salinity fluctuations. In these cases, the application of salinity-independent factory calibration, especially in coarse soils, leads to significant errors in the calculation of θ.

The ε_a_-σ_b_ relationship sensor is strongly linear for all salinity levels. The slope of the linear relationship is salinity dependent and is also affected by the soil type. The Hilhorst model is approximately adequate in measuring σ_p_ in soils with the TEROS 12 sensor.

## Figures and Tables

**Table 1:** Soil Properties.

Soil Type	Clay	Silt	Sand	Dry Bulk Density
	%	%	%	(g/cm^3^)
Sand			100	1.66 ± 0.010
Sandy Loam	16	11	73	1.24 ± 0.010
Loam	19	32	49	1.23 ± 0.011
Clay	48	12	40	1.13 ± 0.012
Sandy Clay Loam	25	12	63	1.26 ± 0.011
Silty Clay Loam	31	49	20	1.11 ± 0.011

**Table 2:** Parameters a and b in the relationship θ-ε^0.5^ for all soil types and salinity levels along with R^2^ values, respectively, and maximum σ_b_ values at saturation.

Soil Type	EC (dS/m)	b	a	R^2^	Max σ_b_ (dS/m)
Sand	0.28	−0.256	0.171	0.973	0.580
	3	−0.288	0.195	0.956	0.940
	6	−0.258	0.194	0.925	1.300
	10	−0.240	0.186	0.938	1.870
Sandy Loam	0.28	−0.226	0.146	0.980	0.563
	3	−0.207	0.144	0.954	1.130
	6	−0.247	0.165	0.967	1.420
	10	−0.181	0.135	0.952	2.140
Loam	0.28	−0.209	0.142	0.961	0.530
	3	−0.202	0.126	0.989	0.930
	6	−0.182	0.132	0.974	1.720
	10	−0.152	0.129	0.869	2.130
Clay	0.28	−0.275	0.143	0.952	1.590
	3	−0.203	0.147	0.932	2.120
	6	−0.210	0.121	0.939	2.550
	10	−0.177	0.109	0.925	2.460
Silty Clay Loam	0.28	−0.299	0.172	0.988	0.440
Sandy Clay Loam	0.28	−0.276	0.155	0.954	0.800

**Table 3:** The RMSE of θ relative to those calculated using manufacturer calibration TEROS 12 and those derived from the CAL procedure.

Manufacturer Calibration				CAL	
Soil Type	Salinity Level	RMSE	Average	RMSE	Average
Sand	0.28	0.056	0.082	0.019	0.026
	3	0.081		0.024	
	6	0.091		0.031	
	10	0.098		0.031	
Sandy Loam	0.28	0.027	0.042	0.014	0.023
	3	0.043		0.027	
	6	0.057		0.023	
	10	0.041		0.028	
Loam	0.28	0.040	0.048	0.025	0.029
	3	0.053		0.026	
	6	0.033		0.020	
	10	0.065		0.046	
Clay	0.28	0.043	0.047	0.033	0.032
	3	0.050		0.030	
	6	0.047		0.031	
	10	0.048		0.035	
Silty Clay Loam	0.28	0.044		0.013	
Sandy Clay Loam	0.28	0.040		0.027	

**Table 4:** Parameters a and b in the relationship ε_a-_σ_b_ for all soil types and salinity levels along with R^2^ values, respectively.

Soil Type	EC	B	a	R^2^
Sand	0.28	1.166	19.360	0.985
	3	2.316	9.344	0.989
	6	2.126	6.604	0.987
	10	1.974	5.729	0.997
Sandy Loam	0.28	1.291	23.018	0.963
	3	2.761	11.938	0.985
	6	2.104	8.600	0.990
	10	1.480	7.625	0.998
Loam	0.28	3.784	25.485	0.997
	3	4.560	12.786	0.993
	6	4.130	8.965	0.990
	10	4.016	7.680	0.992
Clay	0.28	2.296	12.268	0.921
	3	3.100	10.336	0.986
	6	1.880	9.798	0.929
	10	0.683	10.369	0.878
Silty Clay Loam	0.28	4.233	25.740	0.989
Sandy Clay Loam	0.28	3.932	15.462	0.988

**Table 5:** Soil types, values of the conductivity extract’s saturation paste (EC_e_) and values of the electrical conductivity of the pore water (σ_p_) as they have been calculated by the Hilhorst (2000) model.

Soil Type	EC (dS/m)	σ_p_ (dS/m)	EC_e_ (dS/m)
Sand	0.28	5.67	2.770
	3	10.32	
	6	15.48	
	10	17.17	
Sandy Loam	0.28	4.54	1.387
	3	7.40	
	6	10.87	
	10	12.30	
Loam	0.28	3.37	1.880
	3	6.21	
	6	8.54	
	10	10.01	
Clay	0.28	6.34	1.807
	3	7.72	
	6	8.30	
	10	7.26	
Silty Clay Loam	0.28	4.91	2.120
Sandy Clay Loam	0.28	2.92	1.570

## Data Availability

Available upon request.
